# Genetic tracing and topography of spontaneous and stimulated cardiac regeneration in mice

**DOI:** 10.1038/s44161-025-00623-3

**Published:** 2025-03-07

**Authors:** Ilaria Secco, Ana Backovic, Mateusz Tomczyk, Antonio Mura, Gang Li, Francesca Bortolotti, Simone Vodret, Matteo Dal Ferro, Elena Chiavacci, Lorena Zentilin, Gianfranco Sinagra, Serena Zacchigna, Miguel Mano, Mauro Giacca

**Affiliations:** 1 School of Cardiovascular and Metabolic Medicine & Sciences and British Heart Foundation Centre of Research Excellence, King’s College London, London, UK; 2 MRC/BHF Centre of Research Excellence in Advanced Cardiac Therapies (REACT), King’s College London, London, UK; 3 International Centre for Genetic Engineering and Biotechnology (ICGEB), Trieste, Italy; 4 Department of Medical, Surgical and Health Sciences, University of Trieste, Trieste, Italy; 5 Cardiovascular Department, Azienda Sanitaria Universitaria Giuliano-Isontina, University of Trieste, Trieste, Italy; 6 Center for Neuroscience and Cell Biology, University of Coimbra, Coimbra, Portugal

**Keywords:** Cell division, Cell division, Cardiac regeneration, Cardiovascular diseases

## Abstract

Despite recent efforts to stimulate endogenous cardiomyocyte proliferation for cardiac regeneration, the lack of reliable in vivo methods for monitoring cardiomyocyte replication has hindered our understanding of its mechanisms. Thymidine analogs, used to label proliferating cells, are unsuitable for long-term cardiac regeneration studies as their DNA incorporation elicits a damage response, leading to their elimination. Here we present CycleTrack, a genetic strategy based on the transcriptional activation of Cre recombinase from a temporally regulated cyclin B2 promoter segment, for permanent labeling of cardiomyocytes passing through the G2/M phase. Using CycleTrack, we visualized cardiomyocyte turnover in neonatal and adult mice under various conditions, including pregnancy, increased ventricular afterload, and myocardial infarction. CycleTrack also provided visual and quantitative evidence of ventricular remuscularization following treatment with pro-regenerative microRNAs. We identify the subendocardium as a key site of mitotic activity and provide a mode of cardiomyocyte division along their short axis. CycleTrack is a powerful tool to monitor cardiomyocyte renewal during regenerative interventions.

## Abstract (web summary)

Secco et al. develop a genetic tracing method, CycleTrack, to monitor cardiac regeneration. CycleTrack visualizes cardiomyocyte turnover in neonatal and adult mice, after myocardial infarction, and upon stimulation with pro-regenerative microRNAs.

## Main

After damage, such as myocardial infarction, the mammalian heart has a very poor regeneration capacity, unlike teleosts^1,2^ or amphibians^3,4^. This is essentially due to the very low proliferative activity of the survived cardiomyocytes (CMs). Estimates of the basal turnover of human CMs range between 1% per year at age 25 and 0.45% at age 75 (ref. ^5^) and rise only minimally after injury^6^. In contrast to that in adulthood, the mammalian heart undergoes a robust regenerative response after injury in the fetal and perinatal period^7–12^.

Over the past decade, our understanding of the molecular mechanisms that restrict adult versus neonatal CM proliferation has substantially advanced, and a few treatments to unlock the genetic regenerative program in adulthood have been proposed for potential translational application^13^. A common problem that these cardiac regeneration studies have encountered has been the difficulty in obtaining a clear-cut demonstration of CM division after treatment^14–17^. A broadly used strategy to assess CM passage through the S phase is to label newly synthesized DNA using thymidine analogs. The gold standard for studies of cell proliferation has been the use of radiolabeled or stable isotopes of thymidine, in which the chemical composition of the nucleoside is minimally altered. However, radioactive tritiated [^3^H]thymidine, detected by autoradiography, has low spatial resolution and high safety risk, whereas the detection of [^15^N]thymidine stable isotope requires multiple-isotope mass spectrometry, which makes it not suitable for routine use. Thus, most laboratories have taken advantage of the more user-friendly thymidine homologs bromodeoxyuridine (BrdU) or 5-ethynyl-2′-deoxyuridine (EdU), which can be detected with antibodies or using click chemistry, respectively^18^. However, these are not without problems. Antibodies to detect BrdU penetrate whole-mount tissues poorly and require harsh treatment to denature the DNA. EdU detection overcomes these limitations thanks to a simple fluorescent azide-alkyne cycloaddition that can reveal its incorporation in the nucleus. However, both BrdU and EdU have at least three intrinsic limitations. First, they are often used for long-term pulse studies to cumulatively label replicating cells, based on the assumption that these cells can persist long or even divide further while remaining labeled, which is largely unproven. Second, both analogs label the cell nucleus. As CMs are large, cylindric cells, the cell nucleus is not always present in thin histological sections. This leads to an underestimation of the number of labeled cells and, more importantly, forbids the topographical definition of the areas of the myocardium in which CM replication has occurred, as it would be with a replication marker that labels the cell cytoplasm. Lastly, both BrdU and EdU are S-phase markers, and thus they do not provide a reliable estimate of the actual completion of nuclear division.

To overcome these drawbacks, we wanted to develop a system that (1) is not toxic and marks permanently the cells that have replicated, (2) labels cellular mitosis specifically, and (3) permits visualization of the cell cytoplasm and thus provides exact topographical information on where replication has occurred. The system we describe here, which we named CycleTrack, is based on the genetic labeling of cells that traverse the G2/M phase of the cell cycle using a segment of the cyclin B2 (CyB; also known as *CCNB2*) promoter, which is activated with precise temporal control^19,20^, to drive expression of the Cre recombinase, which renders the cytoplasm of replicating CMs permanently fluorescent.

Here, we report on the ineffectiveness of thymidine analogs for the long-term labeling of replicating CMs in vivo and describe how CycleTrack can instead provide a reliable measurement of CM replicative activity in different physiological and pathological conditions.

## Results

### Thymidine analog incorporation is unsuitable for long-term studies

**Extended Data Fig. 1:**
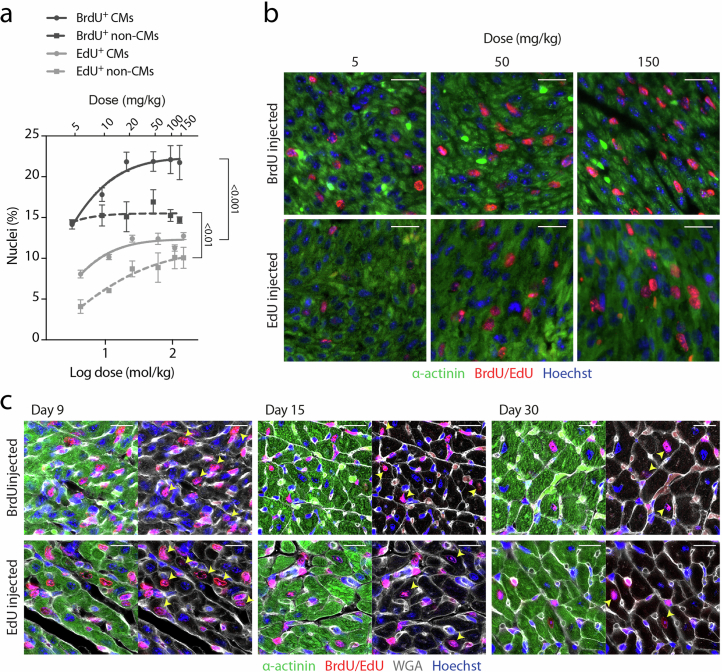
Dose-response study for BrdU/EdU incorporation in mouse hearts. **a**, Quantification of BrdU/EdU-positive nuclei in the heart of mice injected at P1 with indicated doses of the analog and analyzed after 48 hr (n = 3 different animals; 600 to 2000 total CMs per animal; two-way ANOVA with Šidák’s multiple comparisons). Doses are adjusted to mol/kg. Data are displayed as mean±s.e.m. **b**, Representative immunofluorescence images (out of at least 40 different images analyzed) for the indicated doses. Scale bar: 20 μm. **c**, Representative immunofluorescence images (out of at least 40 different images analyzed) used to quantify BrdU- and EdU-positive nuclei. Arrowhead: BrdU/EdU-positive CM. Scale bar: 20 μm. Source data

We revised the suitability of BrdU and EdU labeling to assess CM replication in long-term studies. We started by identifying the minimal dose of the two analogs that was sufficient to mark S-phase cells in neonatal mice. Postnatal day 1 (P1) mice received intraperitoneal injection of the analogs at doses ranging from 5 to 150 mg kg^−1^, followed by assessment of BrdU or EdU incorporation 2 days after injection. Regardless of the dose, BrdU showed higher labeling efficacy than EdU for both CMs and non-CM cells (Extended Data Fig. 1a,b). Incorporation of both analogs plateaued at 20 mg kg^−1^ and above. We concluded that this is the minimal dose of analog that was sufficient to label the maximal number of cells that synthesize DNA in the heart.

**Fig. 1:**
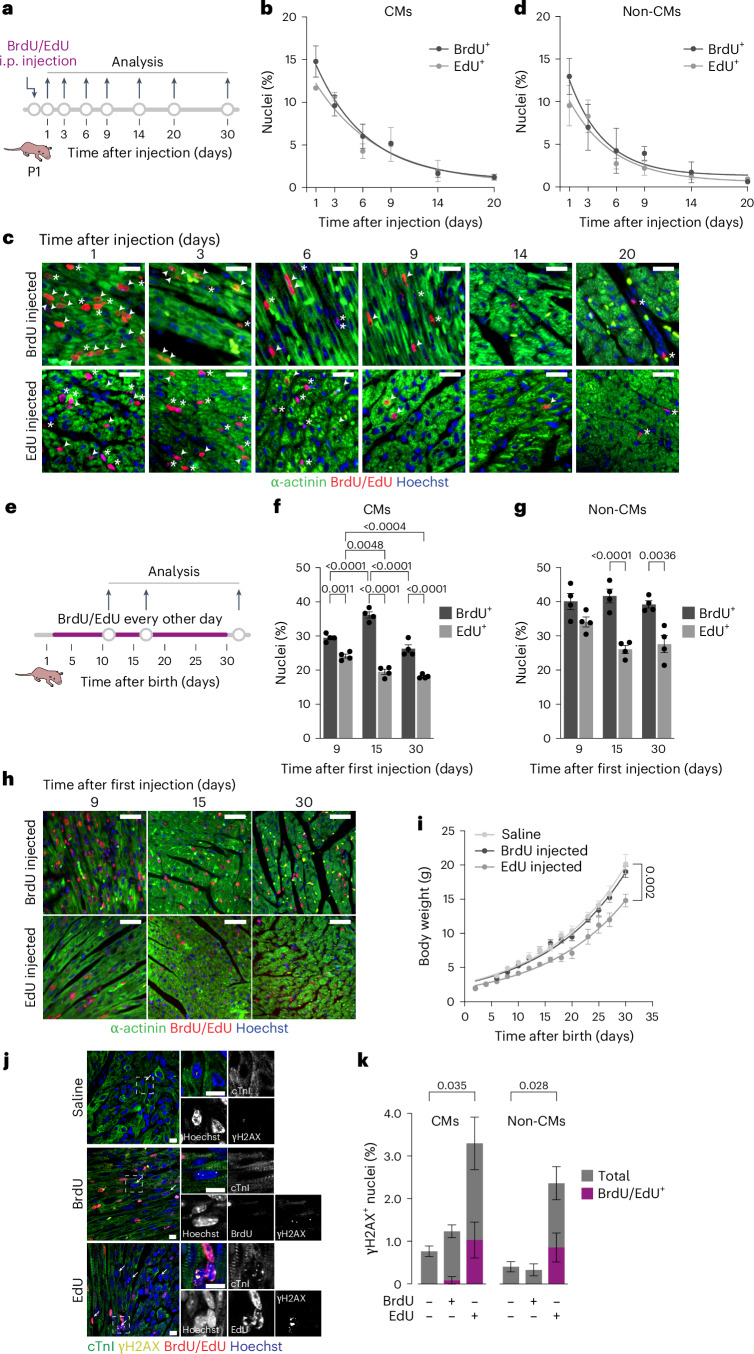
BrdU and EdU incorporation is unsuitable for long-term labeling studies in vivo. **a**, Schematic for 20 mg kg^−1^ BrdU or EdU single injection in P1 mice. **b**–**d**, Representative immunofluorescence images (**c**) and quantification of CMs (**b**) and non-CMs (**d**) for BrdU/EdU incorporation (*n* = 3 mice; 500 to 1,000 total CMs per mouse; two-way analysis of variance (ANOVA) with Šidák’s multiple comparisons: *P* > 0.05). Arrowhead, BrdU/EdU-positive CM; asterisk, BrdU/EdU-positive non-CM. Scale bars, 20 μm. Data are mean ± s.d. **e**, Schematic for 20 mg kg^−1^ BrdU/EdU every other day injection in P2 mice. **f**–**h**, Representative immunofluorescence images (**h**) and quantification of CMs (**f**) and non-CMs (**g**) for BrdU/EdU incorporation (*n* = 4 mice; 400 to 1,000 total CMs per mouse; two-way ANOVA with Tukey’s multiple comparisons). Scale bars, 20 μm. Data are mean ± s.e.m. **i**, Body weight at the time of injection (*n* = 4; repeated measures two-way ANOVA with Dunnett’s multiple comparisons). Data are mean ± s.d. **j**, Immunofluorescence staining of cardiac troponin I (cTnI), γH2AX, and BrdU/EdU with insets. Arrows, γH2AX-positive nuclei. Scale bars, 10 μm. **k**, Quantification of γH2AX-positive and γH2AX-BrdU/EdU double-positive CMs and non-CMs (*n* = 2 mice for saline, *n* = 3 for BrdU/EdU group; 100 to 300 total CMs per mouse; two-way ANOVA with Dunnett’s multiple comparisons). Data are mean ± s.e.m. Source data

Then, we tested the persistence of labeling over time. P1 mice received a single intraperitoneal injection of 20 mg kg^−1^ BrdU or EdU, followed by analysis for 30 days (scheme in Fig. 1a). Labeling was effective at day 1 in the case of both analogs (14.8 ± 1.8% versus 11.7 ± 0.2% labeled CMs, respectively). Still, these percentages gradually declined over time (quantification in Fig. 1b and representative images in Fig. 1c) to progressively become undetectable. The same decay pattern was also observed in non-CM cells (Fig. 1d). Such transient labeling of CMs cannot be attributed to the dilution of incorporated nucleoside due to cell division, as the increase in total CM number in the mouse heart is negligible after birth and CMs do not divide multiple times, either in physiological conditions or upon stimulation^21,22^.

Long-term visualization of cycling cells is commonly achieved by the repeated administration of nucleoside analogs. Thus, we injected newborn mice with 20 mg kg^−1^ of either BrdU or EdU every other day starting from P2, followed by assessment of labeling 9, 15 or 30 days after the first injection (Fig. 1e). The efficiency of repeated labeling was markedly different for BrdU and EdU, with a higher number of apparently cycling CMs detected with the former at all time points. At day 9, the number of labeled CMs was 29.5 ± 0.57% in the BrdU-treated mice versus 24.1 ± 0.59% in the mice injected with EdU. BrdU-labeled CMs increased to 36.2 ± 0.92% after an additional week of BrdU administration but then fell to 26.3 ± 1.1% on day 30 after the first injection. Conversely, EdU failed to show incremental incorporation, with positivity instead declining over time and less than 20% of CMs remaining labeled at day 30 (Fig. 1f,h for quantification and representative images, respectively). Similar considerations also apply to non-CM cardiac cells treated with EdU (Fig. 1g). The group of mice receiving repeated injections of EdU showed signs of long-term toxicity, including impaired growth (Fig. 1i) and evidence of defective homeostasis of high-turnover tissues, such as thinning of the skin and sparse fur.

Long-standing evidence indicates that the incorporation of halogenated pyrimidines, such as BrdU, affects double-stranded DNA break repair and induces genome instability^23^. In addition, EdU is also a substrate for nucleotide excision repair when incorporated into the DNA of replicating cells^24^. Thus, we visualized the levels of phosphorylated H2A histone variant H2AX, a marker of cellular DNA damage^25^. At 24 h after administration of 20 mg kg^−1^ of either BrdU or EdU, the number of total cells with γ-H2AX foci was increased for EdU treatment (representative images and quantification for both CMs and non-CM cells in Fig. 1j,k, respectively). In the mice that received EdU, ~10% of the EdU-positive CMs also scored positive for γ-H2AX (1.03% out of 11.03% of EdU-positive CMs). It is worth noting that less than 30% of all the total γH2AX-positive CMs were also EdU positive. This is consistent with the conclusion that EdU toxicity occurs at levels of incorporation that are even below the threshold required for detection using click chemistry. Analogous considerations also apply to the non-CM cell population. Staining with wheat germ agglutinin assisted in assigning positive nuclei to specific cell types for all these quantifications (Extended Data Fig. 1c).

Altogether, these observations indicate that the administration of thymidine analogs induces the cellular DNA damage response, which triggers cell cycle arrest and activates DNA repair. The progressive loss of labeled cells in vivo and the inability of repeated analog administration to increase the extent of labeling over time can be attributed to either the repair of damaged DNA, with the consequent removal of the label, or the eventual death of the labeled cells. In either case, chemically modified nucleotide incorporation is not a faithful manner to visualize the long-term accumulation of cycling CMs.

### A segment of the CyB promoter is responsive to mitosis

**Extended Data Fig. 2:**
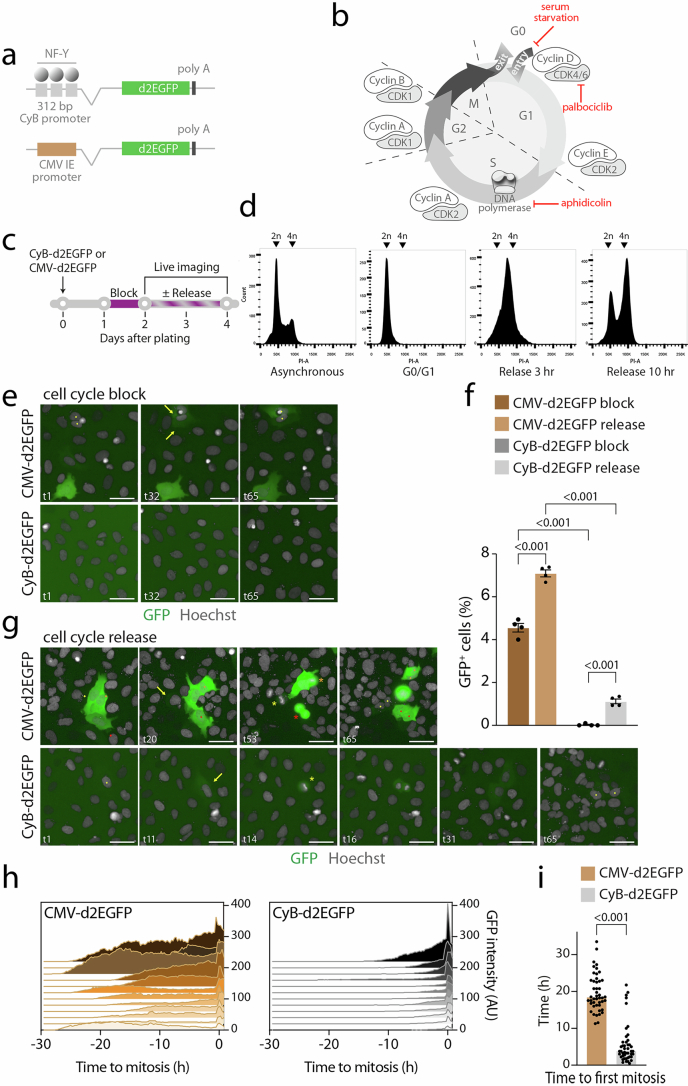
Use of CyB-driven destabilized GFP to study the kinetics of CyB activation. **a**, Schematic of the destabilized GFP expressing constructs. **b**, Diagram of a typical cell cycle with Cyclins and CDKs regulating the progression of each phase. In red: inhibitors used to achieve G0/G1 block in U-2 OS cells. **c**, Outline for the time-lapse recording of U-2 OS cells transfected with either CMV- or CyB-driven d2EGFP. Twenty-four hr after transfection, the cell cycle was blocked for 24 hr and eventually released for additional 48 hr. Cells were transfected with a relatively low amount of plasmid (10 ng/well) to avoid aberrant promoter activation. **d**, Flow cytometry profile of U-2 OS DNA content (propidium iodide, PI) in the indicated conditions (min 4000 cells per condition). 2n: diploid; 4n: tetraploid. **e**-**g**, Quantification (**f**) and representative frames of the time-lapse of U-2 OS cells transfected with either CMV- or CyB-driven d2EGFP blocked in G0/G1 (**e**), or released from the cell cycle block (**g**). Arrow: cell starting to produce d2EGFP; asterisk: mitosis; dot: mother (t1) and daughter cells (t65); n = 4 different replicates; 1600 to 4500 total cells per replicate; two-way ANOVA with Tukey’s multiple comparisons. Scale bar: 50 μm. Data are displayed as mean±s.e.m. **h**, d2EGFP intensity over time of U-2 OS cells transfected with CMV- or CyB-driven d2EGFP after cell cycle release up to mitosis (set as t = 0). (n = 12 from four independent experiments; Y-axis offset: 20 AU). **i**, Quantification of the interval between the appearance of GFP signal and mitosis (n = 46 from four independent biological replicates; Mann-Whitney test, two sided). Individual values and median bars are displayed. Source data

Previous evidence indicates that a short segment of the murine CyB promoter containing 3 CCAAT boxes, which binds transcription factor NF-Y, can detect replicating tissues when positioned upstream of the luciferase gene^19^. We wanted to understand the kinetics of activation of this CyB promoter portion during the cell cycle. We generated a construct containing the part of CyB promoter at −266 to +46 bp (with respect to transcription start site) upstream of the *d2EGFP* gene, which encodes a fast-turnover green fluorescent protein (GFP)^26^ (Extended Data Fig. 2a). This plasmid was transfected into osteosarcoma U-2 OS cells synchronized in G0/G1 by serum starvation and treatment with palbociclib/aphidicolin (experimental schemes in Extended Data Fig. 2b,c and flow cytometry profile for cell synchronization in Extended Data Fig. 2d). A plasmid driving d2EGFP expression under the cytomegalovirus immediate early (CMV-IE) promoter, which is constitutively active, served as a positive control. We found that, in G0/G1 cells, only the CMV promoter was active (Supplementary Video 1 and selected time frames in Extended Data Fig. 2e). When we released the cells from the block by adding serum and removing the drugs, d2EGFP-positive cells started to appear (42.3-fold increase in the number of d2EGFP positive cells versus blocked conditions versus 1.6-fold increase for CMV transfected cells 30 h after release from the block; Extended Data Fig. 2f,g). In these cells released from G0/G1 block, we also monitored the d2EGFP intensity over time leading up to mitosis (Extended Data Fig. 2h) and measured the time to first mitosis. The cells transfected with CyB–d2EGFP showed a significantly shorter median time to first division (3.75 h) compared to the CMV–d2EGFP-transfected cells (18.38 h; Extended Data Fig. 2i). This indicates that promoter activation and mitosis were concurrent for the CyB promoter, while it is stochastic for the CMV promoter. Individual frames from the time lapse in Supplementary Video 1 after release from the block are shown in Extended Data Fig. 2g. Together, these observations indicate that the transcriptional activation of the CyB promoter is concomitant with cell entry into G2 phase followed by mitosis. This specificity of the CyB fragment activation is crucial for the design of our tracing system.

**Fig. 2:**
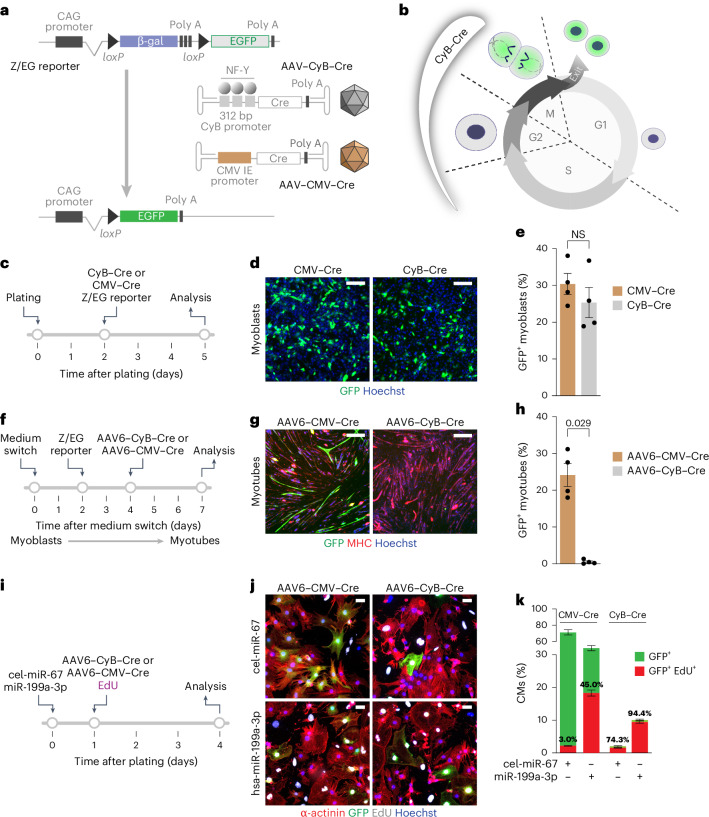
Genetic tracing of mitoses using CycleTrack. **a**, Schematic of the Z/EG reporter for Cre recombinase activation and the AAV vectors carrying either CyB- or CMV-driven Cre recombinase. **b**, Diagram of the cell cycle of a cell with integrated Z/EG reporter transfected or transduced with CyB–Cre. **c**–**e**, Experimental outline (**c**), representative immunofluorescence images (**d**), and quantification (**e**) of C2C12 myoblasts transfected with the Z/EG reporter and either CMV–Cre or CyB–Cre (*n* = 4 replicates; 1,800 to 3,700 total cells per replicate; Mann–Whitney test, two sided). Scale bars, 100 μm. Data are mean ± s.e.m. **f**–**h**, Experimental outline (**f**), representative immunofluorescence images (**g**), and quantification (**h**) of C2C12 myotubes transfected with the Z/EG reporter and transduced with either AAV6–CMV–Cre or AAV6–CyB–Cre (*n* = 4 different replicates; 650 to 1,100 total myotubes per replicate; Mann–Whitney test, two sided). Scale bars, 100 μm. Data are mean ± s.e.m. **i**–**k**, Experimental outline (**i**), representative immunofluorescence images (**j**), and quantification (**k**) of CMs from neonatal Z/EG mice transfected with either cel-miR-67 or hsa-miR-199a-3p and transduced with either AAV6–CMV–Cre or AAV6–CyB–Cre (*n* = 3 replicates for cel-miR-67, *n* = 6 for hsa-miR-199a-3p; 990 to 2,200 total CMs per replicate). Scale bars, 20 μm. Data are mean ± s.e.m. Source data

Next, we generated two plasmids in which either the CyB or the CMV-IE promoter drives the expression of Cre recombinase, flanked by adeno-associated virus (AAV) inverted terminal repeats. In lacZ/EGFP (Z/EG) transgenic mice^27^, Cre activity is monitored using a cassette in which GFP transcription is consequent to the Cre-mediated excision of the β-galactosidase coding sequence (Fig. 2a). When a cell containing an integrated Z/EG reporter is transduced with an AAV vector encoding for CyB-driven Cre, the expression of Cre recombinase is restricted at the G2/M boundary, leading to LoxP site recombination and GFP expression (Fig. 2b). When packaged into an AAV serotype 9 (AAV9) vector, CyB–Cre tracks these mitotic events in CMs only, thanks to the selective tropism of AAV9 for these cells. We named this system for permanent replicating cell labeling CycleTrack. By contrast, when Cre is expressed from the constitutive CMV promoter, both cycling and non-cycling cells are labeled, which can be used to estimate transduction efficiency and Cre efficiency.

To confirm that CycleTrack selectively labels dividing cells, we investigated its activity in the C2C12 cell line, which, depending on serum content, can either be cultured as proliferating myoblasts or be differentiated into resting myotubes (scheme in Fig. 2c). During the process of myogenic differentiation, a decrease in NF-Y subunit A levels causes the loss of functional NF-Y complexes, with a subsequent decline in all its regulated genes, including those involved in cell proliferation^28,29^.

We transfected C1C12 myoblasts with a plasmid containing a Z/EG reporter, identical to that integrated in transgenic Z/EG mice, together with plasmids coding for Cre under the control of either the CyB or CMV promoters. GFP expression driven by either promoter resulted in comparable protein levels in transfected cells, consistent with their replicative properties (30.4 ± 2.9% with CMV–Cre versus 25.4 ± 4.1% of cells with CyB–Cre; Fig. 2d,e). By sharp contrast, when the same experiment was performed in differentiated C2C12 myoblasts using AAV6 vectors (AAV vectors were used here, as myoblasts are refractory to plasmid transfection; Fig. 2f), only AAV6–CMV–Cre was effective, while no GFP-positive cells were detected using AAV6–CyB–Cre (24.1 ± 3.1% with CMV–Cre versus 0.51 ± 0.29% of cells with CyB–Cre; Fig. 2g,h). In these experiments, differentiation was assessed by testing for the levels of myosin heavy chain (MHC) expression.

Next, we tested CycleTrack in primary CMs isolated from the hearts of neonatal Z/EG mice. CMs were transduced with either AAV6–CMV–Cre or AAV6–CyB–Cre and then treated with EdU to label the cells passing through the S phase (experimental scheme in Fig. 2i). When using the CMV–Cre construct, 71.3 ± 0.82% of CMs became GFP positive, which reflects the efficiency of AAV6 transduction. Of these, only 3.0% had also incorporated EdU. When using the CyB–Cre construct, 2.1 ± 0.23% CMs were GFP positive, but of these, 74.3% were also EdU positive. When CMs, before AAV transduction, were stimulated to enter the cell cycle by transfection of miR-199a-3p (a microRNA (miRNA) that our previous work has shown to stimulate CM replication^30–33^), the percentage of EdU-positive cells in the AAV6–CyB–Cre group rose to 10.1 ± 0.78% of total CMs. Of these, 94.4% were also positive for GFP (Fig. 2j,k). By contrast, only 45.0% of the GFP-positive CMs were also EdU positive in the AAV6–CMV–Cre group. The non-targeting miR-67 miRNA from *Caenorhabditis* *elegans* was used as a negative control in these experiments.

Altogether, these data indicate that CycleTrack selectively labels only replicating cells, including CMs. It is worth noting that the cells in which recombination has occurred as well as all their progeny maintain a permanent memory of the mitotic event by remaining stably GFP positive.

### CycleTrack detects mitotic CMs

**Fig. 3:**
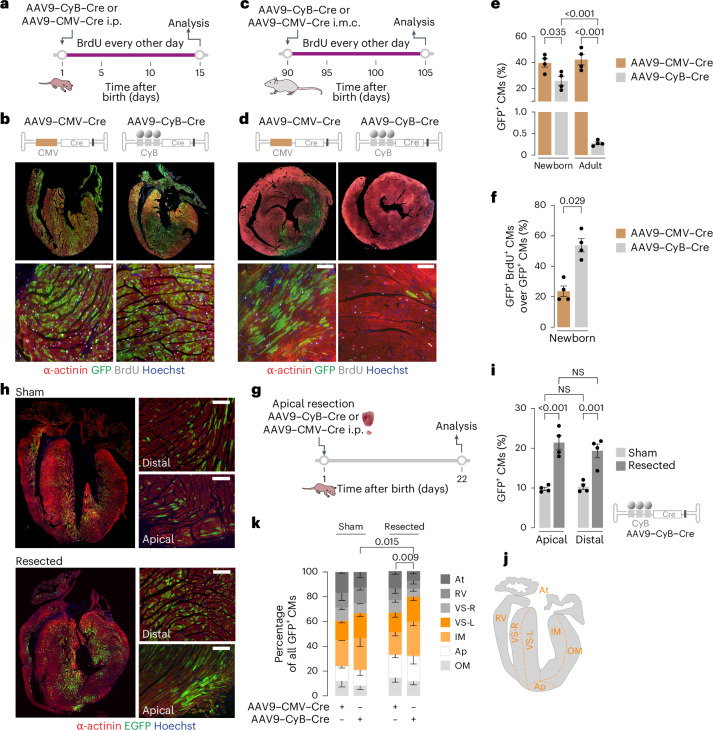
CycleTrack to detect mitotic CMs in neonatal and adult mice and after apical resection in neonatal mice. **a**,**c**, Schematic of neonatal (**a**) and adult (**c**) Z/EG mice injected, respectively, at P1 or P90 with either AAV9–CMV–Cre or CyB–Cre and 20 mg kg^−1^ BrdU every other day. i.m.c., intramyocardial; i.p., intraperitoneal. **b**,**d**–**f**, Representative immunofluorescence images for newborn (**b**) and adult (**d**) mice and quantification of GFP-positive CMs (**e**) and their BrdU/GFP double-positive fraction (**f**) (*n* = 4; one-way ANOVA with Tukey’s multiple comparisons for **e** and Mann–Whitney test for **f**). Scale bars, 100 μm. Data are mean ± s.e.m. **g**–**i**, Schematic (**g**), representative immunofluorescence images (**h**), and quantification (**i**) of Z/EG mice injected with CyB–Cre after eventual resection of the heart apex at P1 (*n* = 4; one-way ANOVA with Tukey’s multiple comparisons). Scale bars, 100 μm. Data are mean ± s.e.m. **j**, Illustration showing the topographic areas for the assessment of GFP-positive CM regional distribution. **k**, Quantification of GFP-positive CM distribution in different regions of the heart (*n* = 3; two-way ANOVA with Šidák’s multiple comparisons). Data are mean ± s.d. Source data

To assess the capability of CycleTrack to monitor the homeostatic CM cycling activity in mice, we developed AAV9 vectors expressing Cre from either the CMV or CyB promoter. When AAV9–CMV–Cre was injected intraperitoneally in neonatal Z/EG mice at P1 (scheme in Fig. 3a), 39.7 ± 3.3% of CMs became GFP positive after a 15-day labeling period (Fig. 3b,e). This percentage is the combined outcome of the efficiency of AAV9 transduction for all CMs irrespective of their replicative state and the duplication of CMs that have previously recombined. When the same experiment was performed with AAV9–CyB–Cre, the percentage of GFP-positive CMs reached 25.7 ± 3.3% (Fig. 3b,e). In this group of mice, the detected Cre activity denotes that at least one event of mitosis had occurred, leading to either cytokinesis or binucleation. Considering that the transduction efficiency in our system was approximately 40%, we concluded that 64.2% of CMs underwent at least one round of division during the 15-day labeling period. This is consistent with the observation by Bergmann and collaborators, who showed that, during the first two post-natal weeks, in mice the total number of CMs increases by approximately 53%^21^.

This scenario was remarkably different after intramyocardial injection of the two vectors in adult Z/EG mice (experimental scheme in Fig. 3c). While the vector expressing Cre from the CMV promoter transduced a large area of the left ventricle (>40% overall transduction in the left ventricle free wall area), the AAV9–CyB–Cre vector was largely inefficient, leading to only a few, sporadic GFP-positive CMs over the 15 day observation period (Fig. 3d,e).

The same groups of neonatal and adult mice also received BrdU every second day to track new DNA synthesis for the entire duration of the experiment. Approximately half of the CycleTrack-positive cells in neonatal mice were also BrdU positive, which is indicative of recent passage through G2/M, while this percentage was markedly lower in CMs receiving CMV-driven Cre (53.54 ± 4.3% versus 23.51 ± 3.4% of cells; Fig. 3f). It is worth noting that the cytoplasmic expression of GFP enabled us to score mitotic events even in those cells in which the nucleus was not visible in the section in analysis.

**Extended Data Fig. 3:**
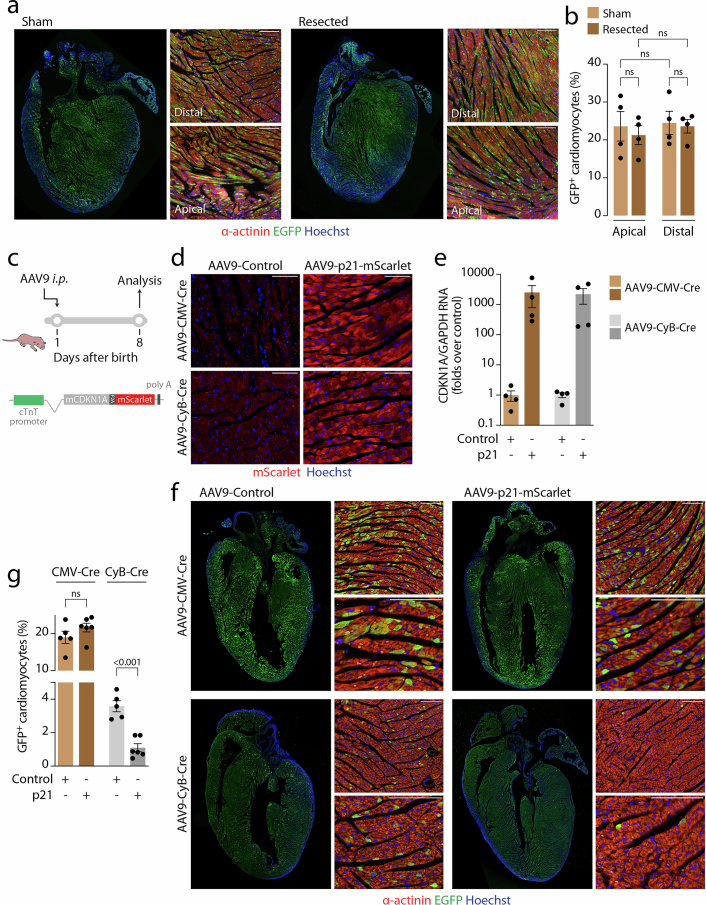
CMV-driven Cre recombinase in neonatal Z/EG hearts after apical resection and CycleTrack response to p21 overexpression in neonatal Z/EG mice. Representative immunofluorescence images (**a**) and quantification (**b**) of Z/EG mice injected with AAV9-CMV-Cre after eventual resection of the heart apex at P1 (n = 4; one-way ANOVA with Tukey’s multiple comparisons). Scale bar: 100 μm. Data are displayed as mean±s.e.m. **c**-**f**, Schematic (**c**) of neonatal Z/EG mice injected at P1 with AAV9-CMV-Cre or CyB-Cre and AAV9-Control of AAV9-p21-mScarlet. Real-time PCR quantification (**e**) of p21 (CDKN1A) normalized on GAPDH and expressed as fold change over AAV9-Control (n = 4; unpaired t-test, two sided). Data are displayed as mean±s.e.m. Representative immunofluorescence images for mScarlet (**d**) and GFP (**f**) and quantification of GFP-positive CMs (**g**) (n = 5; unpaired t-test, two sided). Scale bar: 100 μm. Data are displayed as mean±s.e.m. Source data

To challenge the accuracy of CycleTrack, we intraperitoneally co-injected either AAV9–CMV–Cre or AAV9–CyB–Cre with a control AAV vector or a vector expressing p21 in neonatal Z/EG mice (scheme in Extended Data Fig. 3c). p21 arrests cell cycle progression at both G1/S and G2/M transitions by inhibiting CDK4, CDK6 or CDK2^34^, also in case of exogenous overexpression^35^. In our vector, p21 is driven by the cardiac troponin T (cTnT) promoter and is fused to mScarlet through the self-cleaving peptide P2A (transgene expression at day 7 shown in Extended Data Fig. 3d,e). The co-injection with Cre-expressing vectors ensured that both the tracing and the p21 vectors ended up in the same cells. No effect on Cre recombination was observed in the mice receiving AAV9–CMV–Cre (18.98 ± 1.6% for control and 21.61 ± 1.2% GFP-positive CMs for p21-overexpressing mice), but there was a drastic reduction in CycleTrack-positive cells when mice received the p21 expressing vector (3.58 ± 0.3% versus 1.10 ± 0.2% GFP-positive CMs; Extended Data Fig. 3f,g). These results confirm that our tracing system is accurate and reflects the cell proliferation activity.

### CycleTrack visualizes regeneration after apical resection

The neonatal mouse heart regenerates after partial resection of its apex thanks to the division of pre-existing CMs^36^. To obtain information on the rate of CM proliferation and their topography, we performed apical resection in 1-day-old Z/EG mice, followed by intraperitoneal injection of AAV9–CyB–Cre (scheme in Fig. 3g). Three weeks after the procedure, we detected approximately twice the number of GFP-positive cells in the resected mice, compared to sham-operated mice (Fig. 3h,i). Remarkably, this increase was not restricted to the apical area (9.82 ± 0.40% in sham mice versus 21.45 ± 1.8% of cells in the resected mice) but also extended to distal parts of the left ventricle (10.33 ± 0.68% in sham mice versus 19.36 ± 1.7% of cells in the resected mice). This extension of the CM replicative activity far from the injured area, which is still unexplained in molecular terms, is consistent with similar observations previously obtained using thymidine analog incorporation^36^. Transduction and Cre efficiency, measured as the percentage of GFP-positive cells in mice receiving AAV9–CMV–Cre, did not differ in the sham or resected mice, neither in the proximal nor in the distal areas (Extended Data Fig. 3a,b). To understand whether there were preferential areas in the heart for CM cell cycle activation, we quantified the distribution of recombined cells in each of the sections depicted in the scheme in Fig. 3j, namely, atria (At), right ventricle (RV), right ventricular septum (VS-R), left ventricular septum (VS-L), inner myocardium (IM), apex (Ap), and the outer myocardium (OM) of the left ventricle. We found that the distribution of the GFP-positive cells after CyB–Cre activation was not significantly different from that of CMs in which Cre was activated from the CMV promoter, in both sham and resected mice (Fig. 3k). When considering the left ventricle in its entirety (VS-L + IM + Ap + OM), the prevalence of GFP-positive cells was greater in this area in the resected hearts with CycleTrack compared to both the sham CycleTrack mice and the resected mice receiving CMV–Cre (79.97 ± 3.29% of CMs, 66.62 ± 5.65% of CMs, and 68.28 ± 2.0% of CMs, respectively). Together, these observations indicate that, during regeneration, the expansion of CM proliferation is diffused throughout the left ventricle and not restricted to the apical region and that it is this magnitude of cycling CMs that increases, rather than their regional localization.

### CycleTrack detects CM proliferation during pregnancy

**Fig. 4:**
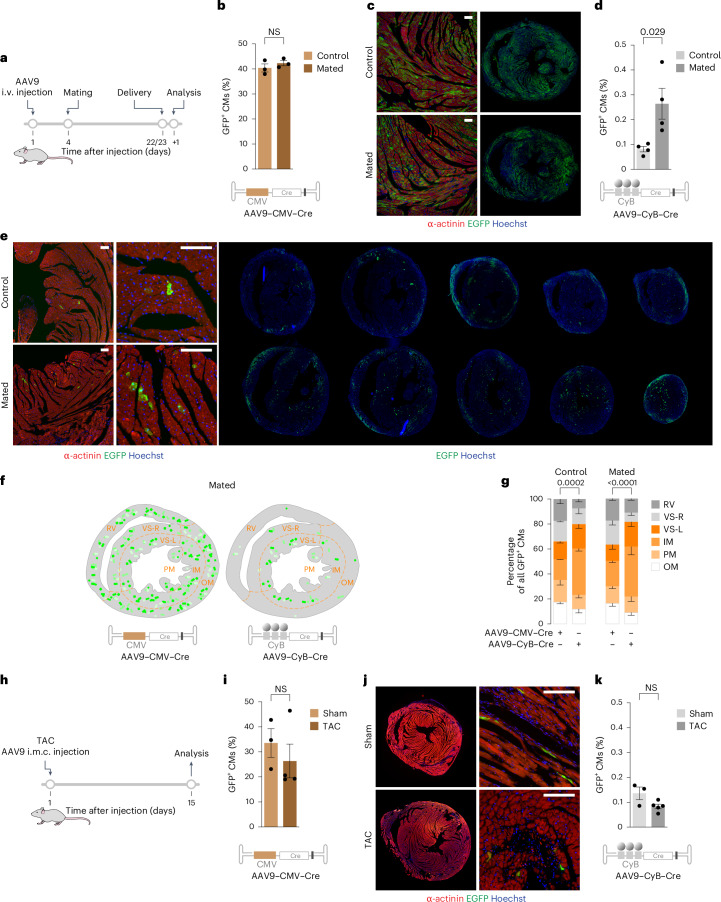
CycleTrack detects CM proliferation during pregnancy and after TAC. **a**, Schematic of Z/EG female mice receiving either AAV9–CMV–Cre or AAV9–CyB–Cre and eventually mated after 4 days. **b**,**c**, Quantification (**b**) and representative images (**c**) of GFP-positive CMs in Z/EG female mice receiving AAV9–CMV–Cre (*n* = 3; Mann–Whitney test, two sided). Scale bars, 100 μm. Data are mean ± s.e.m. **d**,**e**, Quantification (**d**) and representative images (**e**) of GFP-positive CMs in Z/EG female mice receiving AAV9–CyB–Cre (*n* = 4; Mann–Whitney test, two sided). Scale bars, 100 μm. Data are mean ± s.e.m. **f**, Illustration showing the topographic areas for the assessment of GFP-positive CMs regional distribution. **g**, Quantification of GFP-positive CM distribution in different regions of the ventricles (*n* = 3 for CMV–Cre and *n* = 4 for CyB–Cre; two-way ANOVA with Šidák’s multiple comparisons). Data are mean ± s.d. **h**–**k**, Schematic (**h**), quantification (**i**,**k**), and representative immunofluorescence images (**j**) of GFP-positive CMs in Z/EG mice receiving either AAV9–CMV–Cre (**i**) or AAV9–CyB–Cre (**k**) after eventual TAC procedure (*n* = 3 for sham group and *n* = 4 for TAC group; Mann–Whitney test, two sided). Scale bars, 100 μm. Data are mean ± s.e.m. Source data

Our previous work showed that the stimulation of CM proliferation contributes to the increase in cardiac mass observed during pregnancy^37^. We thus wanted to monitor pregnant Z/EG females with CycleTrack. We systemically administered by intravenous injection either AAV9–CMV–Cre or AAV9–CyB–Cre in female Z/EG mice that were eventually mated with males at day 4 after vector administration. All the mice were analyzed 1 day after delivery (scheme in Fig. 4a). The transduction efficiency measured in the group receiving AAV9–CMV–Cre did not change between mated and non-mated mice (42.29 ± 1.0% versus 40.41 ± 1.6% GFP-positive CMs, respectively; Fig. 4b,c). By contrast, in the pregnant female hearts, we detected a 3.2-fold increase in CycleTrack-positive CMs compared to non-mated females (0.26 ± 0.06% versus 0.08 ± 0.01%, respectively; Fig. 4d,e).

**Extended Data Fig. 4:**
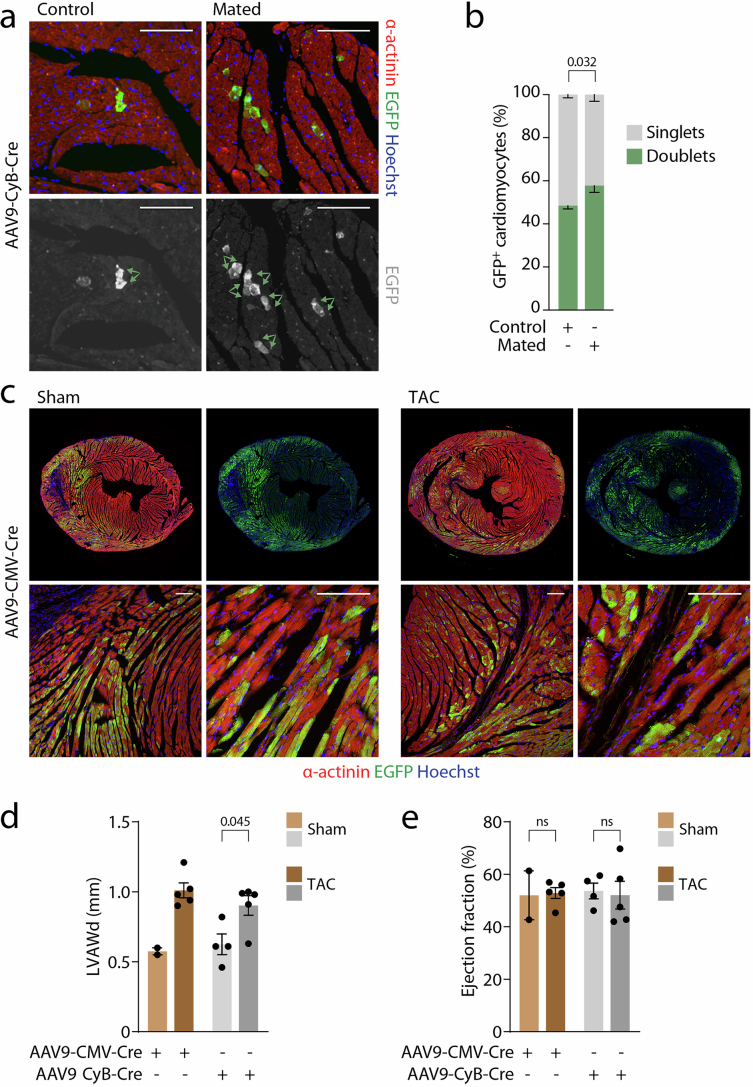
Neighboring CycleTrack-positive CMs during pregnancy and echocardiography assessment of Z/EG mice with TAC. **a**,**b** Representative images and quantification of GFP-positive CMs found as singlets or doublets in Z/EG female mice receiving AAV9-CyB-Cre (n = 4 different animals; Mann-Whitney test, two sided). The green arrows indicate the doublets. Scale bar: 100 μm. Data are displayed as mean±s.e.m. **c**, Representative immunofluorescence images of Z/EG mouse hearts receiving AAV9-CMV-Cre in either sham or TAC condition. Scale bar: 100 μm. **d**,**e**, Echocardiographic measurements of left ventricular anterior wall thickness in diastole (LVAWd) (**d**) and ejection fraction (**e**) at two weeks after sham or TAC procedure. (n = 2-5 different animals; one-way ANOVA with Tukey’s multiple comparisons). Data are displayed as mean±s.e.m. Source data

The presence of neighboring CycleTrack-positive CMs in tissue sections can be used as surrogate evidence of cell division. Thus, we calculated the percentage of GFP-positive CMs that were either isolated (singlets) or neighbored by at least another GFP-positive CM (doublets) in at least four sections from five mice per group. We found that 48.4% of GFP-positive CMs were present as doublets in our control mice and 57.8% after mating (Extended Data Fig. 4a,b). These percentages are very markedly higher than those that could occur by the random transduction of neighboring CMs, which we estimated to be 0.4% in control and 1.3% in mated females, based on the efficiency of CycleTrack labeling (shown in Fig. 4d) and the number of CMs that are in contact with each individual CM (estimated to be 5 from microscopic images). Further considerations on the relevance of neighboring GFP-positive CMs are presented later.

The systemic administration of the vectors allowed us to thoroughly interrogate the localization of the cycling cells in the pregnant female hearts. We thus quantified the spatial distribution of the GFP-positive cells in at least 4 consecutive sections for each of the areas depicted in Fig. 4f, namely, RV, VS-R, VS-L, PM (papillary muscles), IM and OM of the left ventricle. In the case of the control vector, which is not sensitive to the cell cycle, the distribution was even across the different areas, indicating that the AAV uniformly transduces the entire myocardium. Despite this uniform distribution of the AAV vectors, CycleTrack was activated in a remarkably uneven manner, as more than two-thirds of the GFP-positive CMs were found in the region proximal to the left ventricle endocardium and the septum (regions VS-L + IM + PM; 46.96 ± 4.42% GFP-positive CMs for CMV–Cre versus 72.25 ± 4.12% for CyB–Cre for pregnant females; *P* < 0.0001; scheme in Fig. 4f and quantification in Fig. 4g). This uneven distribution of CycleTrack-positive cells was also present for the fewer positive cells found in the control, non-mated mice. These data indicate that, irrespective of the magnitude of CM proliferation, only a subset of CMs enters the cell cycle and that this subset is defined by their localization within the myocardium, possibly reflecting distinct force mechanosensing in different areas of the myocardium.

### CycleTrack is insensitive to CM hypertrophy

Next, we wanted to verify that CycleTrack is uniquely sensitive to cell cycle stimulation and does not respond to cardiac hypertrophy. Z/EG mice were subjected to transverse aortic constriction (TAC) and injected with either AAV9–CMV–Cre or AAV9–CyB–Cre vectors in the anterior left ventricular wall. Two weeks after the procedure (scheme in Fig. 4h), the establishment of hypertrophy was confirmed by echocardiography (Extended Data Fig. 4d), without impairment of cardiac function (Extended Data Fig. 4e). Transduction efficiency was comparable between sham and TAC mice receiving CMV–Cre (33.6 ± 5.7% and 26.4 ± 6.7%, respectively; representative images in Extended Data Fig. 4c and quantification in Fig. 4i). In both groups, the number of CycleTrack-positive CMs was similar to controls without TAC (0.14 ± 0.02% GFP-positive CMs in sham mice and 0.08 ± 0.01% in TAC mice; representative images in Fig. 4j and quantification in Fig. 4k). These findings rule out that CycleTrack is responsive to hypertrophic stimulation.

### Cardiac regeneration after myocardial infarction and miRNA administration

**Fig. 5:**
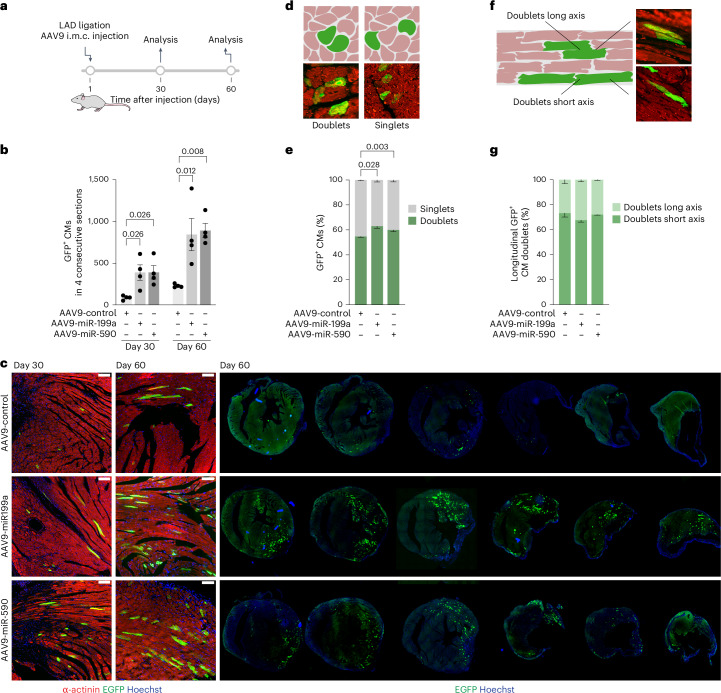
Visualization of cardiac regeneration upon miRNA treatment after myocardial infarction and mode of CM replication. **a**–**c**, Schematic (**a**), quantification (**b**), and representative immunofluorescence images (**c**) of GFP-positive CMs in Z/EG mice receiving AAV9–CyB–Cre and the listed treatments after LAD ligation (*n* = 4; one-way ANOVA with Dunnett’s multiple comparisons, *P* < 0.05 day 60 versus day 30). Scale bars, 100 μm. Data are mean ± s.e.m. **d**,**e**, Illustration and example image (**d**) and quantification (**e**) of GFP-positive CMs found as singlets or doublets at day 60 after infarction (*n* = 4 for control-treated mice, *n* = 3 for miRNA-treated mice; two-way ANOVA with Dunnett’s multiple comparisons). Data are mean ± s.e.m. **f**,**g**, Illustration and example image (**f**) and quantification (**g**) of longitudinal GFP-positive CM doublets likely derived from division along the short or the long axis at day 60 after infarction (*n* = 4 for control-treated mice, *n* = 3 for miRNA-treated mice; two-way ANOVA with Dunnett’s multiple comparisons). Data are mean ± s.e.m. Source data

Finally, we used CycleTrack to visualize and quantify the pro-proliferative effect of two miRNAs (miR-199a-3p and miR-590-3p) that we have previously identified in a screening for miRNAs that induce CM proliferation^30^. Immediately after myocardial infarction, we administered an intramyocardial injection of AAV9–CyB–Cre alone or with either AAV9–miR-199a or AAV9–miR-590 vectors (scheme in Fig. 5a). One month after infarction and treatment, we observed a marked increase in GFP-labeled CMs in the infarct border zone, along with very evident muscularization of the infarcted area (4.5-fold increase in GFP-positive CMs in both miR-199a- and miR-590-treated mice versus control-infarcted mice; quantification of four consecutive sections and representative images in Fig. 5b,c, respectively; *P* < 0.01 for both miRNAs). At day 60 after treatment, the number of GFP-positive CMs was further increased compared to that at day 30. As AAV vectors maintain transgene expression almost indefinitely, this is an indication of an ongoing pro-proliferative effect of the tested miRNAs and of the efficacy of CycleTrack to label proliferating cells cumulatively.

**Extended Data Fig. 5:**
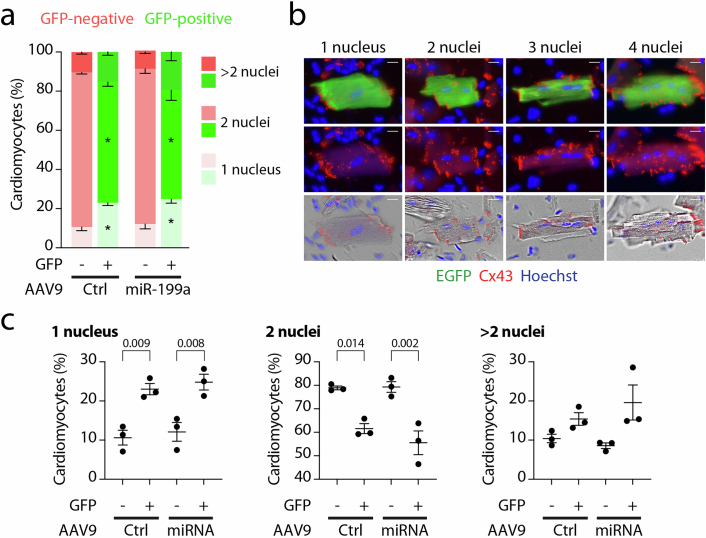
Cardiomyocyte nucleation in CycleTrack-positive population after infarction and miR-199a treatment. Representative immunofluorescence images (**b**) and quantification (**a**,**c**) of the number of nuclei per GFP-negative and GFP-positive CMs isolated from the infarct border zone of Z/EG mice 30 days after LAD coronary artery ligation and injection with AAV9-CyB-Cre and either AAV9-Control or AAV9-miR-199a (n = 3 different animals; 30 to 60 cells per GFP-positive cells per animal, 90 to 110 GFP-negative cells per animal; one-way ANOVA with Tukey’s multiple comparisons). Data are displayed as mean±s.e.m. Scale bar: 20 μm. Source data

To obtain further insights into the relative contribution of cytokinesis versus multinucleation to this increase in CycleTrack-positive cells, we analyzed the number of nuclei in GFP-positive CMs isolated from the injected infarct border zones of Z/EG adult mice 30 days after receiving AAV9–CyB–Cre and either control AAV9 vector or AAV9–miR-199a. The GFP-negative CM population was used to calculate the distribution of nuclei per cell in non-cycling CMs. In this population, we found that 11% of CMs were mononucleated, 79% binucleated, and 10% had 3 or 4 nuclei, irrespective of treatment (Extended Data Fig. 5a–c). In the GFP-positive CM population, in which CycleTrack got activated), the percentage of mononucleated CMs was approximately twofold higher than in GFP-negative cells. This increase occurred in both control-treated mice (as a result of spontaneous CM proliferation consequent to myocardial infarction) and in mice treated with miR-199a. Consistently, the percentage of the binucleated CM population was significantly decreased in the GFP-positive CM population (17% and 23% lower than their respective GFP-negative groups in the control- and miR-199a-treated mice, respectively). As GFP-positive CMs result from a mitotic event, in which the cell becomes irreversibly labeled by CycleTrack, we conclude that GFP-positive mononucleated CMs arise from the cytokinesis of mononucleated CMs, while GFP-positive binucleated CMs form when mononucleated CMs divide their nucleus but do not undergo cell division. As the latter population is decreased, we conclude that cytokinesis of mononucleated CMs is the major event following miR-199a administration.

An additional interesting observation is that treatment with miR-199a, while increasing the total number of GFP-positive cells (Fig. 5b), did not change the CM distribution by number of nuclei. This indicates that this miRNA expands the spontaneous cycling capability of CMs, rather than recruiting, into the cell cycle, CMs that are incapable of dividing spontaneously.

### Mode of CM replication

The visualization of GFP-positive cells using CycleTrack can also provide information on the modality of CM duplication, namely, whether this eventually leads to cytokinesis and how this eventual cell division occurs topologically. As a surrogate for cytokinesis, in each section across the left ventricle, we estimated the percentage of GFP-positive CMs that were either isolated (singlets) or neighbored by at least one other GFP-positive CM (doublets; scheme in Fig. 5d). In principle, singlets are the result of CMs that have passed through the G2/M phase without cell division; doublets have completed cytokinesis. In mice that received the control AAV9 vector, the CycleTrack-positive doublets were 54.73 ± 0.67% of the total GFP-positive cells 2 months after myocardial infarction and treatment. Despite the marked total increase in CMs that had passed through mitosis in the treated mice (Fig. 5b), the percentage of doublets modestly increased to 62.92 ± 1.54% and 60.02 ± 1.50% after AAV9–miR-199a and AAV9–miR-590 treatment, respectively (Fig. 5e). It is worth noting that in all conditions, this observation is an estimate, as our measurements miss CMs in section planes that are immediately above or below the analyzed sections.

The detection of GFP-positive (replicating) CMs in longitudinal planes also offers the possibility to address another long-standing question in cardiac biology, namely, whether CMs divide along their short or long axes. We thus analyzed the percentage of GFP-positive CM doublets that were formed by either CMs that were flanking one another (along the long axis) or CMs that were disposed along their short axis (scheme in Fig. 5f). Interestingly, we found that, in the majority of CMs (>70%), alignment was longitudinal (cell–cell contacts on the short axis), irrespective of whether replication was stimulated by the miRNAs or spontaneous after myocardial infarction (Fig. 5g). The observation that CMs replicate preferentially within existing fibers provides a mechanism by which both physiological and therapeutically induced replication does not induce arrhythmias, as the replication process occurs within an electrical syncytium that is maintained during cell division.

## Discussion

As a method to genetically and permanently mark CMs that undergo mitosis, CycleTrack overcomes several of the limitations of other currently available approaches. The broadly used immunodetection of cell cycle-specific antigens (for example, PCNA or Ki-67) is either for markers of mere cell cycle activation, with no implication for mitosis or cell division or for relatively rare events that are cumbersome to detect and be assigned to CMs in tissue sections (for example, phospho-histone H3 for G2/M or anilin or aurora B in midbodies for cytokinesis). The same considerations also apply to genetic systems such as double EGFP-anilin/αMHC-mCherry^38^ or αMHC-EGFP-anilin^39^ transgenic mice for cytokinesis, or αMHC-FUCCI^40^ for cell cycle state. Despite the elegance of these systems, they only provide snapshot information, similar to antigen immunodetection, and lack the ability to trace the replicative history of the cells.

Our data show that labeling S-phase cells using thymidine analogs is ineffective for long-term studies, as both BrdU and EdU marking are progressively lost in both CMs and non-CM cardiac cells, leading to gross underestimation of CM replicative activity. Loss of labeled cells in neonatal mice cannot be attributed to the dilution of the analogs due to multiple rounds of mitosis, as CMs show a low proliferative rate after birth^21^, parallel to a marked downregulation of cell cycle genes^13^. Most likely, either the thymidine analogs are removed from the DNA or the cells in which they accumulate die. BrdU is known to undergo dehalogenation when integrated into DNA^41^. The resulting uracil base is excised by uracil-DNA glycosylase, leaving an apyrimidinic site that resolves into a nick after apyrimidinic endonuclease action. Similarly, the excision of EdU has also been documented in cultured cells^24^. There is extensive evidence of cytotoxicity of both analogs both in cell cultures^42,43^ and in mice^44,45^. BrdU incorporation alters DNA stability, increasing the risk of sister-chromatid exchanges, mutations, and DNA double-strand breaks^23^; EdU, in addition, causes cell cycle arrest at the G2/M checkpoint^46^ and inhibits essential enzymes involved in nucleoside metabolism^47–49^, eventually leading to cell death. This is consistent with our observation of increased levels of Ser139-phosphorylated histone H2AX in the nuclei of mice receiving EdU, even without detectable levels of the analog by the click chemistry. Our CycleTrack system overcomes several of the limitations of nucleotide labeling. It marks the passage through G2/M phases, ensuring completion of nuclear division rather than relying only on DNA synthesis, which also occurs during DNA repair, polyploidization, or in case of aberrant cell cycle that does not progress to mitosis; it is not toxic; it is stable and allows the visualization of clonal cell expansion, should this occur.

A few transgenic mouse models have been generated and used to trace cycling CMs exploiting DNA recombination, thus providing conceptually similar information to CycleTrack (reviewed in refs. ^50,51^). The only method that proves cytokinesis is the MADM (mosaic analysis with double markers) system^52,53^. This is based on interchromosomal Cre recombination to reconstitute red fluorescent protein (RFP) and GFP genes that, should this occur after the S phase, lead to the emergence of single-labeled cells when recombinant chromatids segregate into different cells during cytokinesis. Given the low probability of these events, this system is problematic at providing a thorough quantification. Two other transgenic models are based on the Ki67 promoter for CM proliferation: the αMHC-MerDreMer-Ki67p-RoxedCre (αDKRC)^54^ and the ProTracer mice^55,56^. Both rely on tamoxifen to activate fate mapping with an efficiency that is either non-disclosed or close to 50% only. Moreover, Ki67 expression is induced in all the phases of the cell cycle except G0, and its activation does not unequivocally mark mitosis^57^. CycleTrack instead precisely traces CM mitoses, as its stringency derives from the specificity of CyB regulation. The mouse CyB promoter contains three CCAAT boxes that are specifically recognized by the trimeric transcription factor NF-Y^58^, which regulates transcription of other cell cycle progression genes^59^ and is the only factor that requires the absolute recognition of all five CCAAT nucleotides^60^. The NF-Y subunit A, responsible for the transactivating activity, is in turn regulated along the cell cycle, both transcriptionally and post-translationally^61^. As a result, CyB expression is restricted to late S and G2/M transition, when only the last checkpoint precludes the cells from completing the cell cycle. In contrast to findings using ProTracer in mice after TAC, where the traced cells were almost 5 times higher than in sham mice^55^, likely as a result of the spurious activation of the Ki67 promoter by hypertrophic stimulation, we did not observe any significant marking increase after TAC in CyB–Cre-injected mice.

Labeling of the CM cytoplasm by GFP expression following CycleTrack activation in Z/EG mice permits visualization of CM replication even in the absence of the CM nucleus in the tissue sections under analysis. This has the additional important advantage of permitting visualization of the myocardial area in which replication has occurred. Using this topographical information, we observed that, after apical resection, a marked increase in CM replication occurs in the entire left ventricle. This suggests that cardiac damage constitutes a global stimulus for regeneration, which could be mediated through either a released factor or a diffusive mechanosensing mechanism. It is worth noting that in both neonatal and adult mice, when proliferation was induced by apical resection or pregnancy, respectively, the distribution of the traced cells did not differ from that of the control mice, but only the magnitude of the proliferation events increased. This indicates that the pro-proliferative stimuli enhance the physiological turnover rate of CMs rather than modifying the characteristics of cells in specific areas.

Cytoplasmic labeling also offers the advantage of providing information on the mode of CM replication. Cells undergoing nuclear division, but not completing cytokinesis, appear as isolated fluorescent cells. By contrast, cells undergoing cell division appear as neighboring cell doublets at the end of the cell cycle, and their reciprocal localization can provide information on the axis at which cell replication has occurred. After miR-199a and miR-590 treatment, the number of labeled CMs increased approximately 4 times at both 30 and 60 days compared to control, infarcted mice. Nonetheless, there was only a modest increase in the GFP-positive doublets compared to control, indicating that miRNA stimulation increases cell proliferation but is capable of also inducing cytokinesis at a rate similar to that occurring spontaneously after myocardial infarction.

The reciprocal localization of neighboring GFP-positive CMs (the products of cell duplication) indicates that, in the vast majority of cases, the division of these cells occurs longitudinally, along their short axis and irrespective of pro-proliferative stimulation by miRNAs. CMs form a functional electrical syncytium by maintaining contacts through the intercalated disc on their short axis^62^. Thus, our observations are in keeping with a model by which a CM divides by maintaining the original intercalated disc connections, making each of the daughter cells already electrically coupled on one of its short axes, similar to what has been reported for adult injured zebrafish heart^63^. This mechanism provides a very plausible explanation of why arrhythmias do not occur during physiological CM replication, for example, in neonatal mice when over 30% of CMs are still actively in the cell cycle^64^.

Finally, CM specificity using the CycleTrack system is based on the use of cardiotropic AAV9 vectors, which only transduce CMs and no other cells in the heart^65^. The use of these vectors also ensures the portability of CycleTrack to any mouse strain or larger animals, provided that an additional AAV vector carrying the recombination template for Cre recombinase is co-administered. In collaboration with the Sadek laboratory, we have recently used such a dual AAV reporter system to track CM proliferative activity in adult CD1 mice after treatment with small molecules^66^.

## Methods

### Plasmid generation

A 312-bp fragment of the mouse CyB promoter was PCR amplified from the pHS4wtB2-Luc vector (a gift from G. Piaggio^19^) adding the sites for MluI and SacII restriction enzymes at the 5′ and 3′ end, respectively. The CyB–MCS (multiple cloning site) backbone was generated by subcloning the amplicon in an AAV2 plasmid containing a MCS downstream of the CMV-IE promoter (AAV–CMV–MCS). The Cre recombinase and the d2EGFP (a gift from D. Rossi; Addgene 26821) sequences were cloned in both backbones using compatible restriction enzymes. The resulting vectors are suitable for both plasmid transfection and recombinant AAV vector production. AAV–CMV–MCS was used to produce control AAV vectors.

The Z/EG reporter was generated by cloning the LacZ (β-galactosidase) and EGFP sequences into a tandem LoxP-containing vector (pCAG-loxPSTOPloxP-ZsGreen, Addgene 51269).

The p21-P2A-mScarlet cassette was synthesized as gBlock (Integrated DNA Technologies) and cloned into pAAV:cTNT::Luciferase (Addgene 69915) through NheI and NotI digestion.

### Production and purification of recombinant AAV vectors

AAV vectors were produced at the AAV Vector Unit Facility at the International Centre for Genetic Engineering and Biotechnology (ICGEB) Trieste. In brief, infectious AAV6 and AAV9 vector particles were produced in human embryonic kidney HEK293T cells by co-transfecting each vector plasmid together with either one packaging plasmid expressing AAV capsid proteins and adenovirus helper proteins (pDF6, PlasmidFactory) for AAV6^67^ or p5E18-VD2/9 (provided by J. M. Wilson) and pHelper (Cell-Biolabs) for AAV9^68^. Viral stocks were obtained by CsCl gradient centrifugation. Recombinant AAV titers, determined by measuring the copy number of viral genomes, were in the range of 1 × 10^12^ to 1 × 10^13^ genome copies per ml.

### Cell lines

C2C12 myoblast cells (ATCC CRL-12772) were cultured in Dulbecco’s modified Eagle medium (DMEM) with 1 g l^−1^ glucose (Gibco) and 20% fetal bovine serum (FBS; Gibco). To induce differentiation, the medium was changed to DMEM with 1% horse serum (Life Technologies). U-2 OS cells (ATCC HTB-96) were cultured in DMEM with 1 g l^−1^ glucose and 10% FBS. Cells were transfected using FuGENE HD transfection reagent (Promega). The optimized ratio of plasmid DNA:lipids was 1:3 and 1:3.5 for C2C12 and U-2 OS, respectively. The complexes were formed in Opti-MEM medium (Gibco). C2C12 myotubes were transduced with AAV6 vectors at a multiplicity of infection of 1 × 10^4^ viral genomes (v.g.) per cell.

### Neonatal mouse ventricular CM isolation

CMs were isolated from the ventricle of P0–P1 Z/EG mice through mincing and 6–8 rounds of 4 min digestion in trypsin–EDTA solution 0.5% (Gibco 1540054) at 37 °C. Most non-CM cells were removed after a 3 h incubation on uncoated dishes. Cells were cultured in DMEM with 4.5 g l^−1^ glucose and 5% FBS, 2 mM l-glutamine (Gibco), 2 μg ml^−1^ vitamin B_12_ (Sigma), 100 U ml^−1^ penicillin, and 100 μg ml^−1^ streptomycin (Sigma) on collagen-coated 96-well plates (Revvity 6055708). Then 50 nM miRNA reverse transfection was performed with Lipofectamine RNAiMAX (Invitrogen) following the manufacturer’s instructions. Human miRNA-199a-3p mimic (Horizon Discovery C-300535-05-0050) and *C. elegans* miRNA-67 mimic (Horizon Discovery CN-001000-01-50) were used. EdU (Invitrogen) was resuspended in DMSO and used at 10 µM concentration. CMs were transduced with AAV6 vectors at a combined multiplicity of infection of 1 × 10^4^ v.g. per cell.

### Cell cycle flow cytometry analysis

U-2 OS cells were collected and fixed in 2% paraformaldehyde (PFA) for 10 min. After one wash with 4% FBS in PBS, cells were incubated with 0.1% Triton X-100 (Sigma), 200 µg ml^−1^ RNase A (Roche 10109142001), and 20 µg ml^−1^ propidium iodide (Invitrogen) in PBS for 30 min at room temperature, filtered through a 70 μm nylon strainer and analyzed at the FACSCelesta Cytometer (BD). Doublets were excluded using forward scatter (FSC) height versus area parameters. Propidium iodide was excited with the 461 nm laser and detected with the 610/20 BP filters. Analysis of the data was performed using the FlowJo v.10 software.

### Cell cycle synchronization and time-lapse imaging

U-2 OS cells were blocked in the G0/G1 phase of the cell cycle by culture in the absence of FBS and treatment with 1 μM palbociclib (PD 0332991 isethionate, Sigma PZ0199) and 3 μM aphidicolin (Sigma A0781) for 24 h. Then 15-min-interval images were acquired for 48 h after the eventual release of the cell cycle block. Images were obtained using Operetta CLS high-content screening microscope (Perkin Elmer) with a Zeiss ×20 objective (numerical aperture (NA) = 0.8) in controlled 37 °C temperature and 5% CO_2_. Hoechst was added at 0.033 µg ml^−1^ 30 min before imaging. Acquisition parameters were the following: binning 2 × 2; Hoechst 20 ms, 5% lamp power; CMV–d2EGFP 10 ms, 4% lamp power; and CyB d2EGFP 20 ms, 20% lamp power. For d2EGFP intensity measurements, 8-bit images were analyzed with the Fiji 1.54f (ImageJ) software, subtracting each frame’s background intensity value and plotting the calculated intensity over time up to mitosis.

### RNA isolation and quantification

The heart tissue was homogenized in QIAzol (Qiagen) in Lysing Matrix D tubes (MP Biomedicals 116913100) at 6,000 r.p.m. for 2 × 15 s. Total RNA was isolated using the miRNeasy Mini Kit (Qiagen); the complementary DNA synthesis was performed with iScript genomic DNA Clear cDNA Synthesis Kit (Bio-Rad), and real-time PCR was run with TaqMan Fast Advanced Master Mix (Applied Biosystems). The expression of *GAPDH* and *CDKN1A* was detected with TaqMan probes (Applied Biosystems Mm99999915_g1 and Mm00432448_m1, respectively).

### Immunofluorescence staining and quantification

For cells, immunostaining was performed using the following primary antibodies: sarcomeric α-actinin (Abcam ab9465; 1:400), MHC (R&D Systems MAB4470; 1:250), and GFP (Abcam ab6556; 1:2,000). Images were obtained using Operetta CLS high-content screening microscope (Perkin Elmer) with a Zeiss ×20 objective (NA = 0.8). A total of 25 fields per well per replicate were imaged and contained approximately 2,500 cells^69^. Cells were analyzed with Harmony 4.9 software (Perkin Elmer) for CM/myotube discrimination and GFP positivity.

For mouse heart tissues, formalin-fixed paraffin-embedded hearts were sectioned at 4 µm in at least 10 different levels >70 µm apart. Sections were de-paraffinized and rehydrated. Antigen retrieval was performed in boiling 0.1 M sodium citrate buffer at pH 6.0 for 20 min. Samples were permeabilized in 0.5% Triton X-100 for 10 min and blocked in 10% goat serum (Abcam) for 1 h. Primary antibodies were diluted in 2% bovine serum albumin (BSA, Roche) and added overnight at 4 °C. The primary antibodies used were the following: sarcomeric α-actinin (Invitrogen MA1-22863; 1:200), cardiac troponin I (Abcam ab47003; 1:250), GFP (Abcam ab6556; 1:2,000), γH2AX (Sigma 05-636; 1:400), BrdU (Abcam ab6326; 1:250), and mCherry (Abcam ab167453; 1:400). BrdU staining was performed as previously described^31^. EdU staining was performed with the Click-iT EdU Alexa Fluor 594 Imaging Kit (Invitrogen C10339) before nuclear staining with Hoechst 33342 (Invitrogen H3570). Single images and scans were obtained using a Nikon Ti microscope and analyzed using the NIS Elements 6.9.0 software.

In the case of EdU and BrdU quantification, random single images were acquired throughout the ventricle. Wheat germ agglutinin (Invitrogen W32466) was added to help with CM nuclei identification, and approximately 500 CM nuclei per animal were considered.

For GFP quantification, large image scans from at least four levels were considered (whole ventricle in case of intraperitoneal or intravenous injection, injected area in case of intramyocardial injection). GFP and α-actinin areas were measured by thresholding the images using the Fiji 1.54f (ImageJ) imaging software and expressed as percentages. For the myocardial infarction study, the number of GFP-positive CMs in four consecutive levels was quantified.

### CM nucleation analysis

Hearts were perfused with 1% PFA and fixed in 4% PFA for at least 2 days. The infarct border zone of the left ventricle was processed as in ref. ^70^. In brief, after overnight incubation in 50% KOH and a wash in PBS 10×, the tissue was gently crushed and vortexed to release the cells, which were pelleted with 1,500 *g* centrifugation and washed two additional washes in 4% FBS in PBS. After permeabilization with 0.5% Triton X-100, anti-GFP (Sigma SAB1305545; 1:100) and anti-connexin 43 (Abcam ab11370; 1:500) were used in 2% BSA for 2 h. After a wash in 4% FBS, the cells were incubated with secondary antibodies (Invitrogen A31571 and A21207) for 1 h in PBS. Cells were then plated with Hoechst (Invitrogen H3570; 1:5,000) and imaged with Operetta CLS high-content screening microscope (Perkin Elmer) with a Zeiss ×20 objective (NA = 0.4) at 6 different planes 5 µm apart. Only intact CMs were considered for the number of nuclei analysis. Non-CM nuclei were excluded based on Z position and size. GFP is present also in the nuclei of a positive CM.

### Animals

All animal procedures were carried out in accordance with Italian and European laws and policies (Directive 2010/63/EU of the European Parliament and of the Council of 22 September 2010 on the Protection of Animals Used for Scientific Purposes) with the approval of ICGEB Animal Welfare Board and the Italian Minister of Health. CD1 mice (Envigo) and Z/EG mice^27^ (The Jackson Laboratory 003920) were housed in the ICGEB Animal Facility in individually ventilated cages under controlled environmental conditions (12 h of light/dark cycle, at approximately 21 °C and humidity 55 ± 10%). The mice were provided with standard laboratory food and water ad libitum.

BrdU and EdU were dissolved in saline at 10 mg ml^−1^ concentration and administered by intraperitoneal injection using an insulin syringe with a 30G needle. For the dose–response study, doses ranging from 5 to 150 mg kg^−1^ were administered in a single injection. For all other studies, repeated injections of 20 mg kg^−1^ of the agent were administered every other day for 30 or 15 days.

### Apical resection in neonatal mice

Heart apical resection was performed according to an established protocol^71^. In brief, Z/EG male and female P1 mouse pups were anesthetized with hypothermia by placing them indirectly on ice for 4 min. During thoracotomy, the heart was exposed, and resection of ∼15–20% of the apical part of the heart with rupture of the left ventricular chamber was performed. Thoracotomy and exposure of the heart alone served as a sham operation. The incisions were closed with 8–0 nylon sutures, and the pups were allowed to recover in a warm environment (heat was provided by a pad and a lamp) until they awakened and resumed normal breathing. The survival rate reached 70% for resected and 98% for sham-operated mice. Resections larger than 20% typically resulted in the pup’s death. Mice received intraperitoneal injection of 1 × 10^11^ AAV9 vector particles with an insulin syringe with a 30G needle.

### Intramyocardial and tail vein injection of AAV9 vectors

Adult, 10- to 12-week-old male and female Z/EG mice were anesthetized by intraperitoneal injection of 100 mg kg^−1^ ketamine and 40 mg kg^−1^ xylazine. Mice were intubated with a 22G cannula that was connected to a mechanical lung ventilator and placed on a temperature-controlled heating pad to prevent loss of body temperature. After confirmation of lack of a withdrawal response to tail and toe pinch, the chest was opened; the heart was exposed by incision of the fourth intercostal space, and the pericardial sac was opened. A maximum of 30 μl of AAV9 vector solution (5 × 10^10^ viral genomes) was administered by direct intramyocardial injection into the free wall of the left ventricle, using an insulin syringe with a 30G needle. Then, the intercostal space and all incisions were repaired with 6–0 nylon monofilament suture. The mouse was extubated to re-establish normal, unassisted breathing.

Adult, 10- to 12-week-old Z/EG mice were administered with AAV9 vectors by intravenous injection via a tail vein. A bolus injection of 1 × 10^11^ of AAV9 viral genomes was administered with an insulin syringe with a 30G needle in a volume not exceeding 4 µl per gram of body weight. Injections were performed on awake mice placed in an appropriate restrainer.

For the experiments in pregnant female mice, AAV vectors were administered 4 days before mating.

### TAC and intramyocardial injection of AAV9 vectors

Permanent aortic arch constriction was performed in 10- to 12-week-old male and female Z/EG mice. In brief, mice were anesthetized and intubated as described earlier. The aortic arch and beating heart were accessed via superior median sternotomy prolonged until the fourth intercostal space. A chest dilatator was used to facilitate the heart and vessel visualization. After gentle separation of the thymus and adipose tissue, constriction of the aortic arch was performed with a 7–0 silk suture loop gauged with a 26G needle. Immediately after constriction, 5 × 10^10^ AAV9 vectors were injected into the left ventricle anterior wall about 1 mm under the left atrium, using an insulin syringe with a 30G needle. The chest and all incisions were closed with 6–0 nylon monofilament sutures, and the animal was moved to a prone position until normal breathing was recovered. Sham-operated mice received all the above procedures except for aortic arch constriction.

### Myocardial infarction

Myocardial infarction was induced in 10- to 12-week-old Z/EG male and female mice by permanent ligation of the left anterior descending (LAD) coronary artery. In brief, mice were anesthetized and intubated, and the heart was exposed as described earlier. A descending branch of the LAD coronary artery was visualized with a stereomicroscope (Leica) and occluded above its bifurcation with an 8–0 nylon suture. Ligation was confirmed by the immediate whitening of a region of the left ventricle that was supplied with blood by the ligated vessel. Next, the mice received 5 × 10^10^ AAV9 vectors by direct intramyocardial injection in the left anterior infarct border zone. Sham-operated mice received all the above procedures except for LAD coronary artery ligation.

### Echocardiography

To evaluate heart function and morphology, transthoracic two-dimensional echocardiography was performed on mice under anesthesia induced with 5% isoflurane and maintained with 2% isoflurane in air, using Vevo 2100 (Visual Sonics) equipped with a 30 MHz linear array transducer. M-mode records in parasternal short axis view were used to measure left ventricle anterior and posterior wall thickness and left ventricle internal diameter at end systole and end diastole. Left ventricle ejection fraction was calculated using Simpson’s method.

### Heart collection and processing

Mice were anesthetized with 5% isoflurane and then killed by injection of 10% KCl solution, to stop the heart in diastole. The heart was excised, briefly washed in PBS and fixed in 4% paraformaldehyde. The tissue was embedded in paraffin and further processed for immunofluorescence staining. A small portion of the apex was snap-frozen for molecular analyses before PFA fixation.

### Statistics and reproducibility

Statistical analyses were performed using GraphPad Prism v. 10.0, and details are included in the figure legends. Statistical *P* values were calculated and reported on graphs. *P* < 0.05 was considered significant. The numbers of mice or replicates for each experiment are reported in the figure legends.

### Reporting summary

Further information on research design is available in the Nature Portfolio Reporting Summary linked to this article.

## Supplementary information

Reporting Summary

Supplementary Video 1 Time-lapse recording of U-2 OS cells expressing CMV- or CyB-driven destabilized GFP. Fluorescent images of U-2 OS cells either blocked in the G0/G1 phase or after the release of the block. The frames shown correspond to images acquired every 15 min between 8 and 24 h after the eventual block release. Upper panels: cells maintained in the cell cycle block; lower panels: cells released from the block. The frames shown correspond to: arrows, cell starting to produce d2EGFP; asterisk, mitosis. Scale bar, 50 μm.

## Source data

Source Data Fig. 1 Statistical source data.

Source Data Fig. 2 Statistical source data.

Source Data Fig. 3 Statistical source data.

Source Data Fig. 4 Statistical source data.

Source Data Fig. 5 Statistical source data.

Source Data Extended Data Fig. 1 Statistical source data.

Source Data Extended Data Fig. 2 Statistical source data.

Source Data Extended Data Fig. 3 Statistical source data.

Source Data Extended Data Fig. 4 Statistical source data.

Source Data Extended Data Fig. 5 Statistical source data.

## Figures and Tables

**Extended Data Fig. 6:**
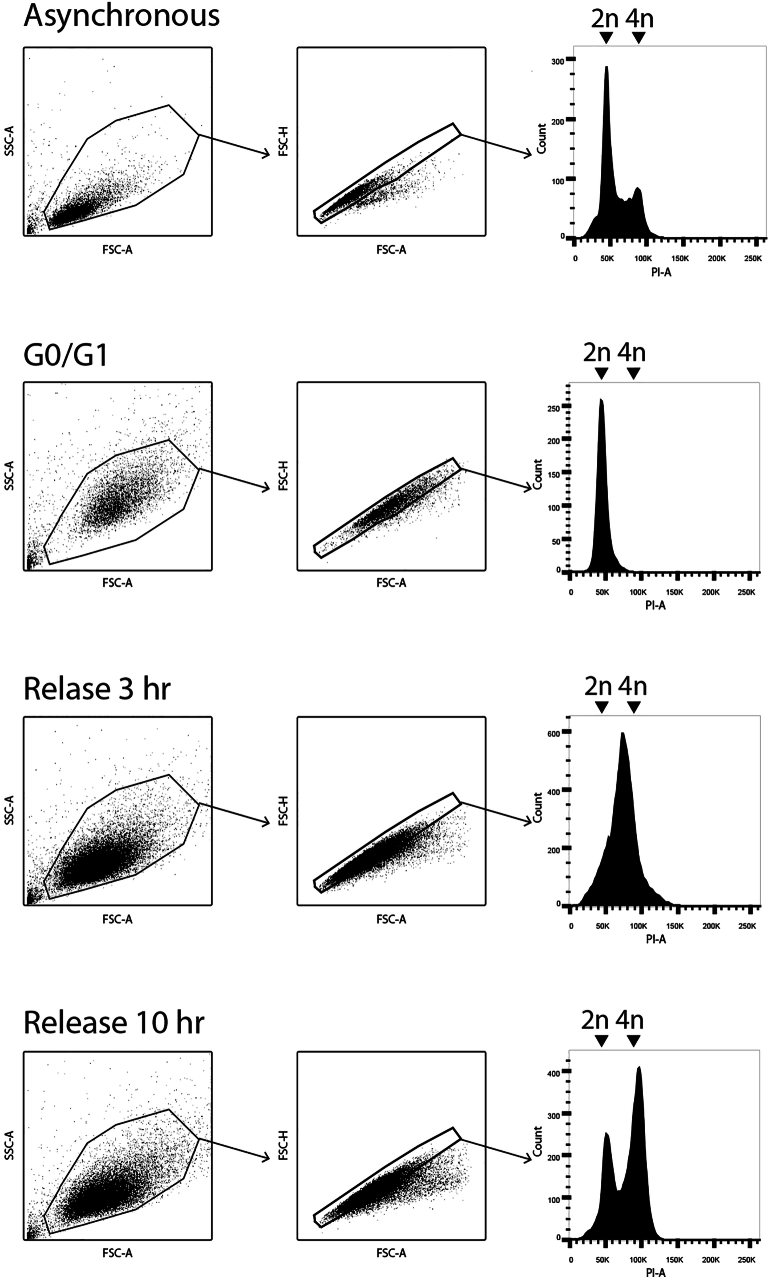
Gating strategy for flow cytometry analysis. The panels refer to the flow cytometry profiles shown in Extended Data Fig. 2d.

## Data Availability

There are no restrictions on data availability. All data are reported in the manuscript main text or its Extended Data information.
