# Supplementary material for: Genetic tracing and topography of spontaneous and stimulated cardiac regeneration in mice
Source: Nat Cardiovasc Res. 2025 Mar 7;4(4):397–411. doi: 10.1038/s44161-025-00623-3 (PMC11994457; doi:10.1038/s44161-025-00623-3)
Supplement: Supplementary file 1 — Reporting Summary [file 44161_2025_623_MOESM1_ESM.pdf]

nature portfolio

Corresponding author(s): Prof. Mauro Giacca

Last updated by author(s): 26/1/25

Reporting Summary

Nature Portfolio wishes to improve the reproducibility of the work that we publish. This form provides structure for consistency and transparency in reporting. For further information on Nature Portfolio policies, see our Editorial Policies and the Editorial Policy Checklist.

Statistics

For all statistical analyses, confirm that the following items are present in the figure legend, table legend, main text, or Methods section.

n/a	Confirmed	
☐	☒	The exact sample size (*n*) for each experimental group/condition, given as a discrete number and unit of measurement
☐	☒	A statement on whether measurements were taken from distinct samples or whether the same sample was measured repeatedly
☐	☒	The statistical test(s) used AND whether they are one- or two-sided<br>*Only common tests should be described solely by name; describe more complex techniques in the Methods section.*
☒	☐	A description of all covariates tested
☐	☒	A description of any assumptions or corrections, such as tests of normality and adjustment for multiple comparisons
☐	☒	A full description of the statistical parameters including central tendency (e.g. means) or other basic estimates (e.g. regression coefficient) AND variation (e.g. standard deviation) or associated estimates of uncertainty (e.g. confidence intervals)
☐	☒	For null hypothesis testing, the test statistic (e.g. *F*, *t*, *r*) with confidence intervals, effect sizes, degrees of freedom and *P* value noted<br>*Give P values as exact values whenever suitable.*
☒	☐	For Bayesian analysis, information on the choice of priors and Markov chain Monte Carlo settings
☒	☐	For hierarchical and complex designs, identification of the appropriate level for tests and full reporting of outcomes
☒	☐	Estimates of effect sizes (e.g. Cohen's *d*, Pearson's *r*), indicating how they were calculated

Our web collection on statistics for biologists contains articles on many of the points above.

Software and code

Policy information about availability of computer code

Data collection	Microscope images: NIS Elements 6.9.0 (Nikon), Harmony 4.9 (Perkin Elmer); Echocardiography: Vevo 2100 (Visual Sonics); Flow cytometry: FACS Celesta Cytometer (BD).
Data analysis	Image analysis: Fiji 1.54f (ImageJ), Harmony 4.9 (Perkin Elmer); Echocardiography: Vevo 2100 (Visual Sonics); Flow cytometry analysis: FlowJo v.10; Statistics: Prism 10.0 (GraphPad).

For manuscripts utilizing custom algorithms or software that are central to the research but not yet described in published literature, software must be made available to editors and reviewers. We strongly encourage code deposition in a community repository (e.g. GitHub). See the Nature Portfolio guidelines for submitting code & software for further information.

Data

Policy information about availability of data

All manuscripts must include a data availability statement. This statement should provide the following information, where applicable:

- Accession codes, unique identifiers, or web links for publicly available datasets
- A description of any restrictions on data availability
- For clinical datasets or third party data, please ensure that the statement adheres to our policy

There are no restrictions on data availability. All data are reported in the manuscript main text or its Extended Data information.

Research involving human participants, their data, or biological material

Policy information about studies with human participants or human data. See also policy information about sex, gender (identity/presentation), and sexual orientation and race, ethnicity and racism.

Reporting on sex and gender	n/a
Reporting on race, ethnicity, or other socially relevant groupings	n/a
Population characteristics	n/a
Recruitment	n/a
Ethics oversight	n/a

Note that full information on the approval of the study protocol must also be provided in the manuscript.

Field-specific reporting

Please select the one below that is the best fit for your research. If you are not sure, read the appropriate sections before making your selection.

☒ Life sciences ☐ Behavioural & social sciences ☐ Ecological, evolutionary & environmental sciences

For a reference copy of the document with all sections, see nature.com/documents/nr-reporting-summary-flat.pdf

Life sciences study design

All studies must disclose on these points even when the disclosure is negative.

Sample size	Sample size for each experiment is reported in the Figure Legends. Sample size for animal experiments was determined by power analysis, considering an effect size estimated from prior experience in the same experiments in our laboratory. Sample size for in vitro studies was determined from our prior experience in similar experiments and/or analogous reports from the literature.
Data exclusions	No data were excluded.
Replication	All the experiments were performed in three or more indipendent biological replicates. All the biological and technical replicas were successful.
Randomization	Mice were randomly assigned to each experimental/control group. The analysed mice were litter mates and sex-matched whenever possible. For experiments in vitro, samples were randomly allocated to treatment.
Blinding	For all the experiments, including echocardiography studies and image analyses, the investigators were blinded to the treatment.

Reporting for specific materials, systems and methods

We require information from authors about some types of materials, experimental systems and methods used in many studies. Here, indicate whether each material, system or method listed is relevant to your study. If you are not sure if a list item applies to your research, read the appropriate section before selecting a response.

Materials & experimental systems

n/a	Involved in the study	
☐	☒	Antibodies
☐	☒	Eukaryotic cell lines
☒	☐	Palaeontology and archaeology
☐	☒	Animals and other organisms
☒	☐	Clinical data
☒	☐	Dual use research of concern
☒	☐	Plants

Methods

n/a	Involved in the study	
☒	☐	ChIP-seq
☐	☒	Flow cytometry
☒	☐	MRI-based neuroimaging

Antibodies

Antibodies used	Mouse monoclonal anti-Sarcomeric alpha-actinin (clone EA-53) (Abcam, Cat #ab9465; 1:400, and Invitrogen #MA1-22863; 1:200);

Antibodies used	mouse monoclonal anti-Myosin heavy chain (clone MF20) (R&D Systems #MAB4470; 1:250), rabbit polyclonal anti-GFP (Abcam #ab6556; 1:2000), rabbit polyclonal anti-Cardiac troponin I (Abcam #ab47003; 1:250), mouse monoclonal anti-phospho-Histone H2A.X (Ser139) (clone JBW301) (Sigma #05-636; 1:400), rat monoclonal anti-BrdU (clone BU1/75 (ICR1)) (Abcam #ab6326; 1:250); rabbit polyclonal against mCherry (Abcam #ab167453; 1:400).
Validation	The specificity of the antibodies was provided by the manufacturers - please see specific web site information for each of the antibodies used.

Eukaryotic cell lines

Policy information about cell lines and Sex and Gender in Research

Cell line source(s)	Primary neonatal cardiomyocytes were isolated from male and female Z/EG mice.<br>C2C12 cells were purchased from ATCC (CRL-12772); U-2 OS cells were purchased from ATCC (HTB-96); HEK-293T cells were purchased from ATCC (CRL-3216).
Authentication	Primary neaonatal cardiomyocytes were identified with specific immunostaining. No cell line authentication was performed as directly purchased from ATCC.
Mycoplasma contamination	Cells were tested negative for mycoplasma.
Commonly misidentified lines<br>(See ICLAC register)	None of the cell lines are listed in the ICLAC database.

Animals and other research organisms

Policy information about studies involving animals; ARRIVE guidelines recommended for reporting animal research, and Sex and Gender in Research

Laboratory animals	CD1 mice (Envigo) and Z/EG mice (The Jackson Laboratory #003920) were housed in the ICGEB Animal Facility under controlled environmental conditions, at a 12-hour light and 12-hour dark cycle, with ad libitum access to water and food. Experiments in adult animals were performed in in 10-12 week old animals.
Wild animals	The study did not involve wild animals.
Reporting on sex	For primary cardiomyocyte isolation, apical resection, myocardial infarction and transverse aortic contriction, mixed sexes of Z/EG mice were used.. For BrdU and EdU studies, mixed sexes of CD1 mice were used. For pregnancy, female Z/EG mice weree used. Sex was assigned anatomically by the ICGEB Animal Facility staff. The number of animals used in each study is reported in Figure Legends. No sex-based analysis was performed as it was not the goal of the study.
Field-collected samples	The study did not involve field-collectes samples.
Ethics oversight	All animal procedures were carried out in accordance with Italian and European laws and policies (Directive 2010/63/EU of the European Parliament and of the Council of 22 September 2010 on the Protection of Animals Used for Scientific Purposes) with the approval of ICGEB Animal Welfare Board and the Italian Minister of Health.

Note that full information on the approval of the study protocol must also be provided in the manuscript.

Plants

Seed stocks	n/a
Novel plant genotypes	n/a
Authentication	n/a

Flow Cytometry

Plots

Confirm that:

☒ The axis labels state the marker and fluorochrome used (e.g. CD4-FITC).

☒ The axis scales are clearly visible. Include numbers along axes only for bottom left plot of group (a 'group' is an analysis of identical markers).

☒ All plots are contour plots with outliers or pseudocolor plots.

☒ A numerical value for number of cells or percentage (with statistics) is provided.

Methodology

Sample preparation	U-2 OS cells were harvested and fixed in 2% PFA for 10 min. After one wash with 4% FBS in PBS, cells were incubated with 0.1% Triton X-100 (Sigma), 200 µg/mL RNase A (Roche #10109142001) and 20 µg/mL propidium iodide (PI, Invitrogen) in PBS for 30 mins at room temperature, filtered through a 70 µm nylon strainer
Instrument	FACSCelesta Cell Analyzer (BD)
Software	FACSDiva (BD) for data collection, FlowJo X (Becton & Dickinson and Company) for data analysis.
Cell population abundance	Not applicable as the cell line was analysed for DNA content only.
Gating strategy	A gate was drawn around the population of interest excluding cell debris (SSC-A/FSC-A: 10/32k) and doublets (FSC-A: 40-190k/FSC-H: 20-130k).

☒ Tick this box to confirm that a figure exemplifying the gating strategy is provided in the Supplementary Information.
